# Multivariate analysis of early surgical management factors affecting posttraumatic penoscrotal avulsion injury: a level I trauma center study

**DOI:** 10.1186/s12894-020-00763-7

**Published:** 2021-01-07

**Authors:** Min Ji Kim, Dong Hwan Lee, Dong Ha Park, Il Jae Lee

**Affiliations:** Department of Plastic and Reconstructive Surgery, Ajou University School of Medicine, 164, World cup-ro, Yeongtong-gu, Suwon, 16499 Republic of Korea

**Keywords:** Penoscrotal avulsion, Trauma, Wound management, Quality improvement, Wound and injury

## Abstract

**Background:**

To conduct an accurate evaluation of patients presenting with posttraumatic penoscrotal injuries, and to formulate a treatment algorithm based on this assessment.

**Methods:**

We conducted a retrospective chart review study. Patients with penoscrotal defects admitted to our level I trauma center from 2017 to 2019 were evaluated. The Braden scale score was used for wound evaluation and the Korean patient classification system (KPCS) was used for assessment of severity. Univariate and multivariate analyses were performed for potential risk factors associated with early surgical management.

**Results:**

In total, there were 58 male patients, and the average Braden scale score was 12.08 ± 2.54, with the scrotum (36.20%), and the penile shaft (32.76%) being popular sites for injuries. The wounds requiring surgical treatment were 20.68% (*n* = 12), with local flaps (33.33%) being most commonly used. The significant predictors of advanced wounds which required surgical treatment were old age (*p* = 0.026, odds ratio [OR] 8.238), orthopedic combined injuries (*p* = 0.044, OR 1.088), intubation (*p* = 0.018, OR 9.625), restraint (*p* = 0.036, OR 0.157) and blood transfusion (*p* < 0.001, OR 2.462).

**Conclusion:**

In multiple trauma patients, penoscrotal defects caused by high-speed trauma are an important matter of concern. Specifically, patients with combined skeletal injuries or requiring respiratory care were prone to advanced wounds. We proposed a five-category algorithm to manage such patients, which included severity of the patient’s condition, respiration, hemodynamic status, comorbidity, and immobilization. Additionally, inter-departmental cooperation and active intervention by plastic surgeons is needed for the comprehensive treatment of such injuries.

*Trial registration* This study was performed in line with the principles of the Declaration of Helsinki. The study and all its protocols were approved by the institutional review board of Ajou Medical Center (approval no. AJIRB-MED-MDB-17-254). The need for informed consent was waived by the institutional review board of our hospital due to the retrospective design of the study.

## Background

In multiple severe trauma patients, high-speed mechanism injuries in accidents can cause penoscrotal defects. The importance of this region for the body’s urologic and sexual functions is well known, and the preservation of the structure and function of the penoscrotal region is integral to the patient’s quality of life [1]. In some disease involving penoscrotal defects such as Fournier’s gangrene, the physician has to focus on wound management, often necessitating early referral to a reconstructive specialist [2]. However, severely trauma patients admitted to a level I trauma center differ from patients suffering from a single disease. Recently, cancer-related mortality has decreased by 20% (1991–2009); however, trauma-related mortality has increased by 24% (1990–2010) [3, 4]. These patients are managed according to a structured resuscitation protocol. Furthermore, these multiple trauma patients may present with an edematous perineum, hiding a penoscrotal avulsion injury. In such critical conditions, such wounds that are not immediately obvious, can easily be overlooked even by plastic surgeons.

For the management of penoscrotal avulsion injuries, a comprehensive evaluation of the patient’s status is needed. Surgical reconstruction of a penoscrotal lesion is not simple. The goal is to provide wound healing, adequate function, and an acceptable appearance [5]. To date, recent studies have underestimated such injuries; thus, information regarding these injuries is scarce. The purpose of this study was to provide an accurate evaluation of trauma patients with penoscrotal injuries and to propose a treatment algorithm based on this assessment.

## Methods

We retrospectively recruited patients who were admitted to our level I trauma center, with trauma-related penoscrotal defects between 2017 and 2019. The following demographic information was collected; age, sex, cause of injury, combined injuries, management protocols including respiratory care, intubation, nutrition, restraint, range of motion, and whether or not they were alive at presentation. The wound characteristics were evaluated according to severity, anatomical lesion, defect size, management technique, operative details, and complications. The Braden scale score was used for wound evaluation, and the KPCS (Korean patient classification system) was used for the assessment for patient severity [6–9]. The Braden Scale assesses risk using six different risk factors: sensory perception, the ability to respond meaningfully to pressure-related discomfort; moisture, defined as the degree to which skin is exposed to moisture; activity, degree of physical activity; mobility, defined as the ability to change and control body position; nutrition, usual food intake pattern; and friction and shear [6, 10, 11]. The Braden scale is widely used to evaluate the risk of sores. It has several components to enable various aspects of wound evaluation. However, it can be used to evaluate other wounds apart from sores. KPCS consisted of 12 areas, 50 nursing activities, and 73 items (factors for general nursing care) [12]. It was developed based on the Workload management system for nurses (WMSN) of the USA. There are many reports of its validity and reliability; therefore, this classification has been included in the basic tools used in tertiary hospitals in Korea [13–15]. Wound dimension was assessed using ImageJ (National Institutes of Health, USA) [16]. For each photographic image, measurements were calibrated using pixels at the margin of the wound comparing with the entire genital area. The wound size was calculated using the percentage of the total genital area according to pixel count. Statistical analysis was performed using Statistical Package for the Social Sciences (SPSS) for Windows version 18.0 (SPSS Inc., Chicago, IL, USA). Data are presented as means ±2 standard deviations. Statistical significance was accepted at *p* < 0.05. Factors that predicted surgically demanding and advanced wounds were identified through a univariate analysis. A surgically demanding wound is defined as an open wound that cannot be expected to heal completely with conservative dressing treatment. Such wounds are expected to require surgical intervention to enable healing. Advanced wounds are a sub-category of surgically demanding wounds. These wounds require surgical treatment, but the focus of treatment is tissue reconstruction. Therefore, simple wounds that require only debridement are excluded from this category. Variables with a *p*-value< 0.05 were included in the multivariate analysis. Backward-stepwise logistic regression analysis was performed to identify risk factors for trauma-related advanced penoscrotal defects. The need for informed consent was waived by the Institutional Review Board because of the retrospective nature of the study. However, photographic authorization and release consent were obtained from all participants, including parental consent for adolescent patients. This study strictly adhered to Declaration of Helsinki principles.

## Results

A total of 58 patients were included in this retrospective study. All the patients were male and had an average age of 59.72 years (range, 40–78). Mean follow-up period was 13.74 months (range, 5.5–22). Intensive care unit patient severity, as assessed by KPCS, had mean values of 4.41 ± 1.22. Wound severity assessed by Braden scale had an average of 12.08, and high-risk patients for whom scale was lower than 12 were 65.52%. The most common cause of injury was a fall, followed by traffic accidents, each contributing to 37.93 and 22.41% of the injuries, respectively. Wound extension was classified on the basis of anatomical sites, and scrotum and shaft were the most commonly injured sires, at 36.20 and 32.76% respectively [17]. On comparing with the total genital area, the involved penoscrotal defect portion was 55.80 ± 30.45% (Table 1).

**Table 1:** Patient demographics

Variable	Values
No. of subjects	58
Age (years)^a^	59.72 ± 19.25
Sex (n)	
M	58
Severity^b^	4.41 ± 1.22
Braden scale^a^	12.08 ± 2.54
Braden scale (n, %)	
≤ 18	38 (65.521)
Cause of injury (n, %)	
Fall	22 (37.93)
Traffic accident	13 (22.41)
Motorcycle accident	9 (15.51)
Rolling injury	4 (6.90)
Crushing injury	5 (8.62)
Others	5 (8.62)
Site of lesion (n, %)	
Glans	2 (3.44)
Corona	1 (1.72)
Foreskin	8 (1.38)
Shaft	19 (32.76)
Root	7 (12.07)
Scrotum	21 (36.20)
Size of defect (% of total genital area)^a^	55.80 ± 30.45
Follow up months^a^	13.74 ± 8.20

^a^Mean ± 2SD

^b^Korean Patient Classification System for Nurses

Comorbidities were classified into nine areas: brain, orthopedic, spine, facial, vascular, lung, intraorgan, and soft tissue comorbidities and acute respiratory distress syndrome. The most common combined injury was orthopedic injury (65.62%), followed by intraorgan injury (37.93%), and spine injury (34.48%). Extensive soft tissue injuries were found in 20.69%, but these injuries did not involve genital wounds. Among 58 patients, 75.85% survived, however, 15 patients died in spite of active resuscitation at a professional trauma center. The rate of ventilator assistance was 44.83, and 91.38% of the patients received a blood transfusion during the hospital stay. Some hemodynamic indicators were evaluated; the collected mean hemoglobin level was 11.41 ± 1.80 and the mean creatinine level was 1.22 ± 0.83 (Table 2).

**Table 2:** General comorbidities and hemodynamic status in the study population

Variable	Values
Combined injury (n, %)	
Brain injury	15 (25.86)
Orthopedic injury	38 (65.62)
Spine injury	20 (34.48)
Facial bone injury	7 (12.07)
Vascular injury	15 (25.86)
Lung injury	19 (15.51)
Intraorgan injury	22 (37.93)
Extensive soft tissue injury (except genital)	12 (20.69)
Acute respiratory distress syndrome	10 (17.24)
Intubation (n, %)	26 (44.83)
Absolute bed rest (n, %)	8 (13.79)
Restrain (n, %)	24 (41.37)
Total parenteral nutrition (n, %)	25 (43.10)
Transfusion (n, %)	53 (91.38)
Initial hemodynamic status ^a^	
Hb	11.41 ± 1.80
Albumin	3.32 ± 0.69
Bun	17.42 ± 8.87
Creatinine	1.22 ± 0.83
Survival (n, %)	44 (75.86)
Expire (n, %)	15 (25.86)

^a^Mean ± 2SD

**Fig. 1:**
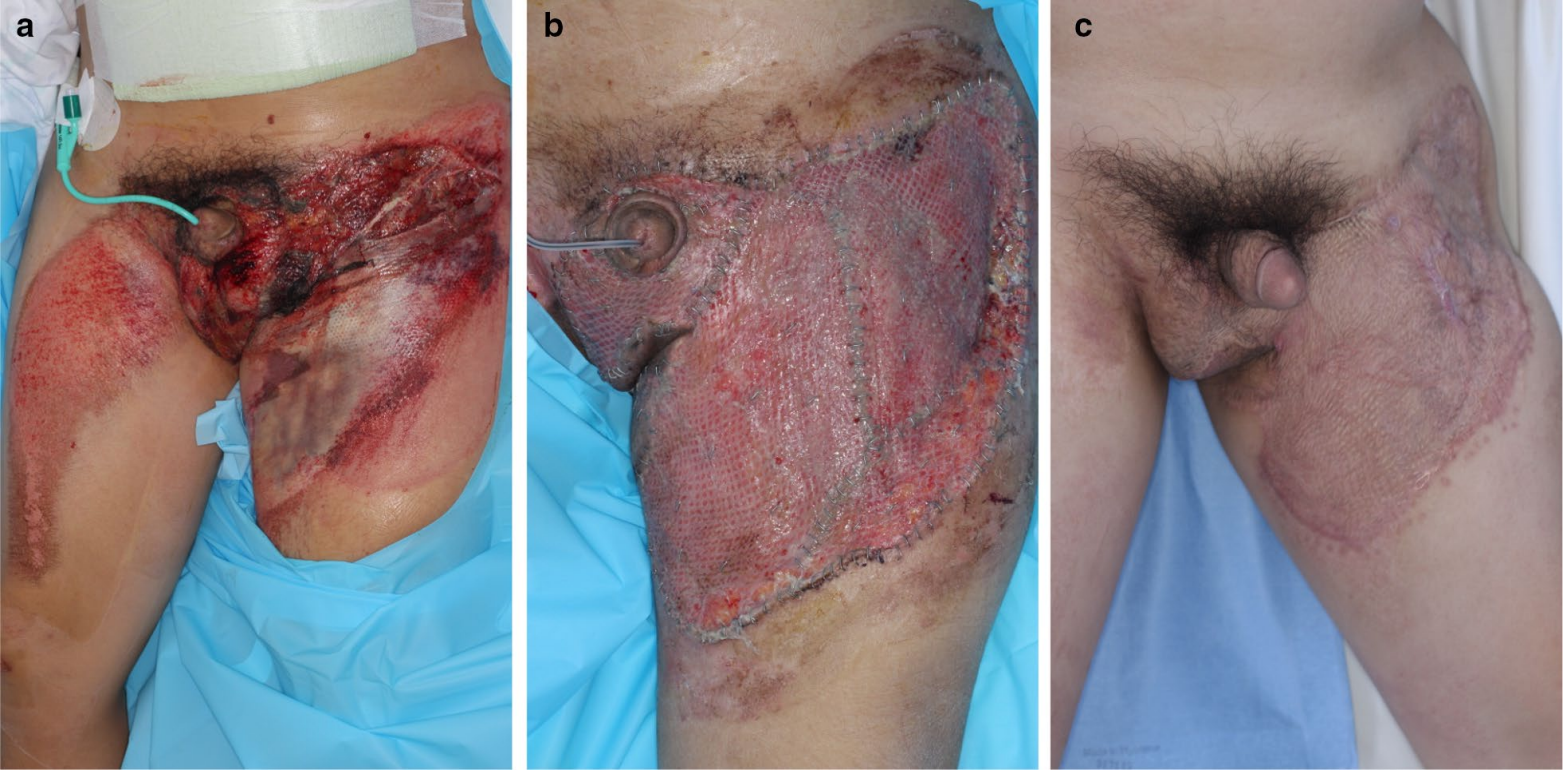
Representative case of a penoscrotal injury where successful surgical reconstruction was achieved. **a** The 36-year-old male pedestrian with no underlying conditions was hit by a vehicle and admitted for soft tissue injury involving the scrotum and thigh, an ankle fracture, and a pelvic bone fracture. He was transfused for massive blood loss, but not intubated. The urologist confirmed that the wound did not invade the testicles and he had no problems with sexual function and urination. **b** After several debridements, he underwent a split-thickness skin graft. **c** One year later, he had discomfort due to scar contracture in his inguinal area, and the scar contracture was released with a Z-plasty. According to the five-category algorithm, he had orthopedic injury, required restraint, and transfusion—a total of three factors that could signify an advanced wound

**Fig. 2:**
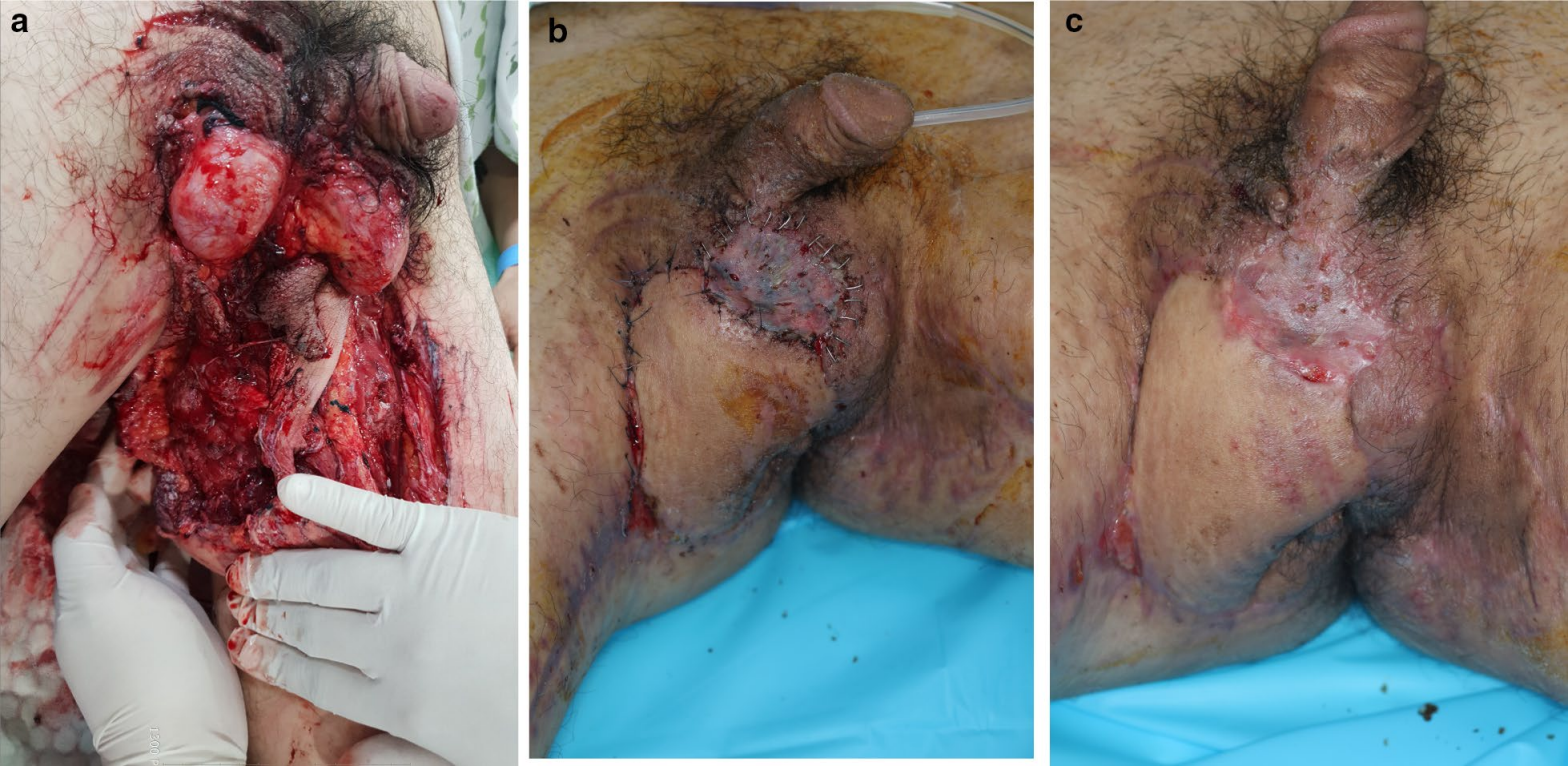
Representative case of penoscrotal injury where successful surgical reconstruction was achieved. **a** A 34-year-old man with no underlying disease admitted for soft tissue injuries including the scrotum, bilateral thighs, and perineum, because his thigh rolled into a rolling machine. His vital signs were stable. There was no testicular and anal injury. **b** After several debridements, a medial circumflex artery perforator-based local flap rotation, and split-thickness skin grafting were performed. **c** After 1 month, the wound was well healed. According to the five-category algorithm, he had intubation, required restraint, and transfusion—a total of three factors that could signify an advanced wound

Among the 58 patients, 12 received surgical wound management. The remaining 47 patients were treated with conservative management, which included aseptic dressing, negative pressure vacuum dressing, and growth factor ingredient medication-assisted dressing. The operative options ranged from debridements and local flaps to skin grafts and free flaps (Figs. 1, 2). The most common modality used was local flap in 33.33% of patients, followed by debridement in 25%, and free flap in 25%. Severe complications were not noted, and only minor complications, including partial necrosis, were noted (Table 3).

**Table 3:** Outcome of surgical, non-surgical management of penoscrotal defect

Variable	Values
Conservative management (n, %)	47 (81.03)
Surgical management (n, %)	12 (20.68)
Operative option	
Debridement	3 (25.00)
Local flap	4 (33.33)
Skin graft	2 (16.67)
Free flap	3 (25.00)
Surgical complication	
Infection	–
Partial necrosis	3 (25.00)
Total necrosis	–

Univariate analysis identified several clinical variables associated with the development of a surgically demanding advanced wound. Age, Braden scale, orthopedic injury, intraorgan injury, extensive soft tissue injury, intubation, absolute bed rest, restraint, transfusion, hemoglobin, blood urea nitrogen, and survival status were found to be associated with advanced wounds. Multivariate logistic regression analysis of these variables showed that old age (*p* = 0.026, OR 8.238), orthopedic combined injury (*p* = 0.044, OR 1.088), intubation (*p* = 0.018, OR 9.625), restraint (*p* = 0.036, OR 0.157), and transfusion (*p* < 0.001, OR 2.462), were significant predictors of advanced wounds which would require surgical treatment (Table 4).

**Table 4:** Variables associated with surgical management of trauma-induced penoscrotal defect

Variables	Univariate analysis			Multivariate analysis	
	Odds ratio	***p***-value	Odds ratio	95% CI	***p***-value
Age	1.019	0.006^a^	8.238	0.000, 10.944	0.026^a^
Severity	0.727	0.210	–	–	–
Braden scale	0.548	0.047^a^	2.462	1.497, 4.049	0.877
Cause of injury	1.205	0.057	–	–	–
Brain injury	0.339	0.142	–	–	–
Orthopedic injury	0.460	0.048^a^	1.088	0.008, 1.932	0.044^a^
Spine injury	0.374	0.145	–	–	–
Facial injury	0.693	0.683	–	–	–
Vascular injury	1.019	0.978	–	–	–
Lung injury	0.577	0.394	–	–	–
Intraorgan injury	1.231	0.018^a^	3.087	0.010, 9.724	0.402
Extensive soft tissue injury	1.933	< 0.001^a^	6.772	1.258, 13.578	0.210
ARDS	2.617	0.284	–	–	–
Intubation	2.903	0.021^a^	9.625	1.466, 63.549	0.018^a^
Absolute bed rest	6.429	0.028^a^	1.657	0.147, 3.488	0.344
Restrain	0.253	0.042^a^	1.157	0.028, 0.888	0.036^a^
Total parenteral nutrition	5.040	0.155	–	–	–
Transfusion	2.201	0.001^a^	2.462	1.497, 4.049	< 0.001^a^
Hb	0.229	0.001^a^	1.050	0.733, 1.502	0.791
Albumin	1.821	0.285	–	–	–
BUN	1.254	0.020^a^	0.818	0.691, 0.969	0.351
Creatinine	0.002	0.060	–	–	–
Expire	0.039	0.022^a^	2.007	0.261, 3.988	0.157

^a^ Statistically significant; *ARDS* acute respiratory distress syndrome, *BUN* blood urea nitrogen

## Discussion

The development of high-speed machines and an advanced building industry has changed the presentation of trauma patients and it leads to associated industrial accidents as well as traffic accidents. Such trauma can involve every part of the body, including the penoscrotal area. Owing to their concealed anatomical locations, such injuries can go easily undetected, further delaying treatment. There are few comments on the mechanism of development of penoscrotal injuries in a trauma patient, besides a direct injury of the perineal area from the trauma. In rotation machines, the capturing force of the machine creates torsion and leads to traction of the area [18, 19]. In falls, direct trauma to the falling side causes friction burns which lead to soft tissue injuries. If the scrotal skin loss is less than 50%, it can often be closed immediately after the trauma [19]. The depth of injury can involve damage to the cavernous bodies, spongy body, or testes [20]. Following blood loss, infection and lymphedema can lead to the development of an irreversible wound.

**Fig. 3:**
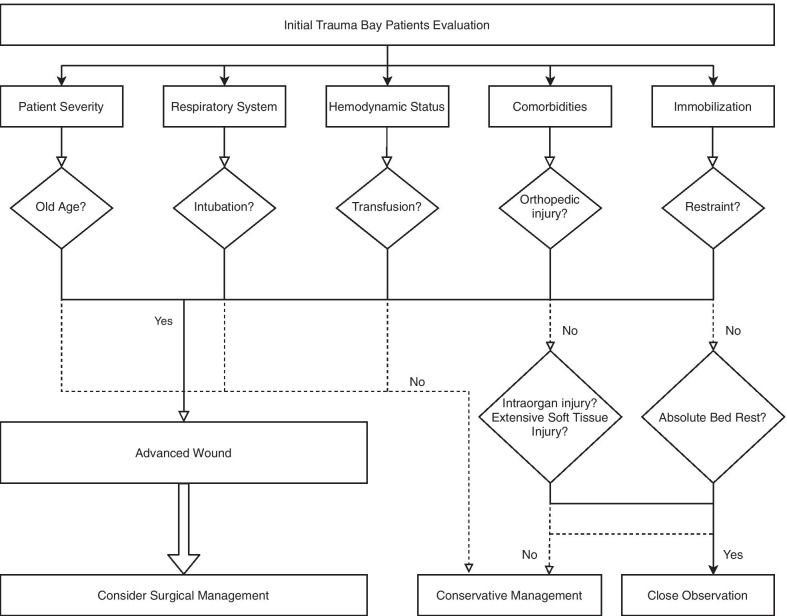
Treatment algorithm for posttraumatic penoscrotal injury

In this study, our final proposal was an assessment algorithm for trauma-related penoscrotal avulsion injury (Fig. 3). We composed a five-category algorithm, based on our multivariate clinical risk evaluation. The categories were patient severity, respiration, hemodynamic status, comorbidities, and immobilization. In this algorithm, the main factors that contribute to advanced penoscrotal injury are composed of patient stability, immobilization, and oxygen insufficiency. Orthopedic injury and restraint contribute to immobilization, and this can lead to delayed wound healing and wound deepening. Insufficient respiration and blood loss contribute to oxygen insufficiency, which leads to wound necrosis and aggravation of the edema. Through this algorithm, we want to emphasize that such patients should not simply be referred to the plastic surgeon. Initially, the critical care team should get involved with the wound evaluation and discuss management with other teams. The categorized component starts from the initial patient evaluation. Initially in the emergency room, a comprehensive wound approach is required for successful reconstruction. During the patient’s hospital stay, there might be further possibilities for the critical care team to get involved. However, our study stated that when the algorithm is first applied, we simply need to check the wound status to determine whether the wound is likely to progress into an advanced wound.

The details of the treatment algorithm are described below. Firstly, in multiple trauma patients, the initial hemodynamic status should be assessed. Various laboratory findings and hemodynamic indicators help us decide whether resuscitation treatments should be started immediately or later. Hemodynamic instability should be considered first; if the patient is persistently unstable, stabilization without active wound management should be considered. Massive blood loss usually requires transfusion; thus, integration of transfusion management should be considered. The next step is the severity of the patient’s condition, which should be considered with respect to their age. Respiratory care with intubation, in our experience, increased the risk of penoscrotal injury with a highly advanced wound status; thus, active surgical management should be considered. In multiple trauma patients, their additive injury status leads to further problems. Thus, a categorized evaluation is needed. In particular, combined orthopedic injuries were associated with high rates of advanced penoscrotal wounds which required special attention. Moreover, intraorgan injuries and extensive soft tissue injuries are often associated with advanced penoscrotal wounds; however, no statistical significance was noted in our study. Finally, based on these five categories of evaluation in trauma patients, we aim to detect penoscrotal wounds earlier and start active interventions.

Why is the early diagnosis of surgical prone penoscrotal defects in trauma patients important for reconstruction? Extensive penoscrotal defects ultimately require surgical reconstruction. However, anatomically the lesion penoscrotal area, there is a presence of circumferential muscle and its flexible skin texture has to be dealt with in a very particular attention. Especially erectile function should be reproduced via functional reconstruction. These days, sensate flap reconstruction is possible, hence one should not delay a referral. In our study, the most common surgical option was a local flap. The range of local flaps includes skin advancement using inguinal to upper thigh skin, local fasciocutaneous flaps, and musculocutaneous flaps. The next step for a successful reconstruction is choosing a reconstructive option. This decision is best made after the serial debridement. Once we follow the penoscrotal defect from the initial status without infection, the debrided necrotic tissue can help the surgeon make the decision. Once healthy granulation tissue is observed, we can apply the skin graft, which works best on the scrotum and shaft. As these areas have very thin epithelium this vacuum-assisted treatment is very helpful. However, complex anatomical lesions and wounds extended to the inguinal area are limited with respect to formation of granulation tissue. In this case, we used local flap advancement by using the surrounding abdominal or thigh soft tissue. However, in cases of exposed tendon or vessel, a free flap is required. Functionally and aesthetically pleasing reconstructions are now possible. However, while choosing an appropriate method of surgical reconstruction, we need to take the patient’s overall status into consideration.

The limitations of our research are as follows. Owing to the nature of a level I trauma center, the severity of the patients’ condition is relatively high, which could have contributed to the higher mortality seen in our study compared to those in other studies. The resuscitation rates such as respiratory resuscitation and transfusion rates were also very high. However, the mortality itself was not directly associated with the severity of the penoscrotal injury. Therefore, there was a limitation in the association between the nature of the trauma center itself with the severity of the wounds. Through this study, we hope that the discovery algorithm used to detect penoscrotal injuries in traumatic patients, will now include more than just an assessment of wound complexity.

## Conclusions

In multiple trauma patients, due to the requirement of comprehensive management, penoscrotal defects caused by trauma are prone to going undetected. However, the functional and psychological importance of this area demands definitive attention and care from the physician, especially for patients with combined skeletal injuries or those on respiratory care who are likely to have advanced wounds warranting surgical treatment. We proposed a five-category algorithm to manage penoscrotal injuries in trauma patients. Inter-departmental cooperation and active intervention by plastic surgeons is needed for comprehensive treatment. We hope the proposed algorithm will help in the early detection of trauma-related penoscrotal injuries and promote early referral for their active surgical or non-surgical management.

## Data Availability

The datasets used and/or analysed during the current study available from the corresponding author on reasonable request.

## Abbreviations

KPCS: Korean patient classification system
